# Radiological Insights into Sacroiliitis: A Narrative Review

**DOI:** 10.3390/clinpract14010009

**Published:** 2024-01-03

**Authors:** Asma’a Al-Mnayyis, Shrouq Obeidat, Ammar Badr, Basil Jouryyeh, Saif Azzam, Hayat Al Bibi, Yara Al-Gwairy, Sarah Al Sharie, Giustino Varrassi

**Affiliations:** 1 Department of Clinical Sciences, Faculty of Medicine, Yarmouk University, Irbid 21163, Jordan; 2 Faculty of Medicine, Yarmouk University, Irbid 21163, Jordan; anaobeidat1@gmail.com (S.O.); ammarinb@hotmail.com (A.B.); bassiljoureyah978@gmail.com (B.J.); saifazzam2000@gmail.com (S.A.); hayatbb20000@gmail.com (H.A.B.); yaraalghwairy@gmail.com (Y.A.-G.);; 3 Paolo Procacci Foundation, 00193 Roma, Italy; giuvarr@gmail.com

**Keywords:** sacroiliitis, MRI, SIJ, inflammation, radiography

## Abstract

Sacroiliitis is the inflammation of the sacroiliac joint, the largest axial joint in the human body, contributing to 25% of lower back pain cases. It can be detected using various imaging techniques like radiography, MRI, and CT scans. Treatments range from conservative methods to invasive procedures. Recent advancements in artificial intelligence offer precise detection of this condition through imaging. Treatment options range from physical therapy and medications to invasive methods like joint injections and surgery. Future management looks promising with advanced imaging, regenerative medicine, and biologic therapies, especially for conditions like ankylosing spondylitis. We conducted a review on sacroiliitis using imaging data from sources like PubMed and Scopus. Only English studies focusing on sacroiliitis’s radiological aspects were included. The findings were organized and presented narratively.

## 1. Introduction

Sacroiliitis is an inflammatory process that affects the sacroiliac joint (SIJ). It may appear acutely or along the course of chronic diseases [1]. The SIJs have an important role in transferring loads between the lumbar spine and the lower limbs and providing support for the upper body [2,3]. Inflammation and mechanical disfunctions are the two underlying mechanisms for any sacroiliac joint abnormality [3]. It has been reported that 25% of cases of lower back pain is related to the SIJ, of which this pain may stem from various causes such as sacroiliac joint dysfunction (SIJD), stress, pregnancy, trauma, lumbar fusion surgery, bone graft near the sacroiliac joint, and others [3]. The only way to detect structural changes accompanying sacroiliitis is the use of various imaging modalities. Often plain radiography is utilized, especially for its advantageous features. It is considered a safe option, given its low radiation exposure, and it is also cost effective and readily accessible [1]. Concerns have arisen due to its limitation of being a two-dimensional technique to evaluate three-dimensional structures. Therefore, other modalities have been considered such as Computed Tomography (CT) scans, Magnetic Resonance Imaging (MRI), and nuclear medicine scans (bone scans) [4,5,6,7].

The treatment of sacroiliitis spans a spectrum, ranging from conservative and osteopathic interventions to more interventional approaches such as radiofrequency denervation and joint fusion [3]. Notably, recent technological advancements have showcased the potential for artificial intelligence networks to detect radiographic sacroiliitis with expert-level precision [8].

In this narrative review, we will discuss sacroiliitis by describing the radiological anatomy of the SIJ, the etiologies of sacroiliitis, its clinical presentation, diagnostic imaging modalities, radiological criteria, the management of sacroiliitis, and any future directions in this field.

## 2. Materials and Methods

In order to examine the clinical and radiological characteristics of sacroiliitis, we performed a narrative review. The principal aim of the research was to comprehend and synthesize numerous imaging and clinical observations linked to this condition.

Using the following keywords, we searched through a number of reputable sources, including PubMed, Scopus, and Web of Science: “sacroiliitis”, “radiological features”, “imaging”, “X-ray”, “MRI”, and “CT scan”, “Ankylosing Spondylitis”, and “Sacroiliac joint”.

Studies that were published in English, mainly addressed sacroiliitis and its radiological manifestations, made use of any imaging modalities—such as MRIs, CT scans, or X-rays—or that offered precise and comprehensive depictions of radiological results were all considered for inclusion. Studies that were not primarily focused on sacroiliitis or its radiological aspects, opinion pieces, letters to the editor lacking original data, conference abstracts, or published in languages other than English without a trusted translation were all omitted.

**Figure 1:**
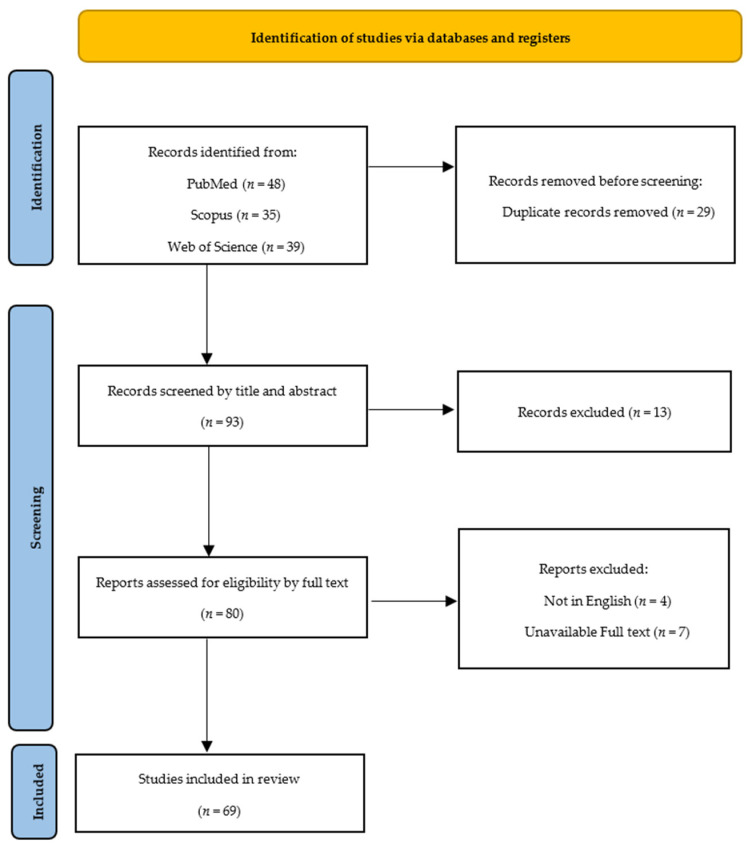
Preferred Reporting Items for Systematic Reviews and Meta-Analyses (PRISMA) flowchart.

Relevant data about the radiological characteristics of sacroiliitis were collected, organized into categories, and presented following the Preferred Reporting Items for Systematic Reviews and Meta-Analyses (PRISMA) guidelines (Figure 1) [9].

## 3. Results and Discussion

### 3.1. Anatomy of the Sacroiliac Joint

**Figure 2:**
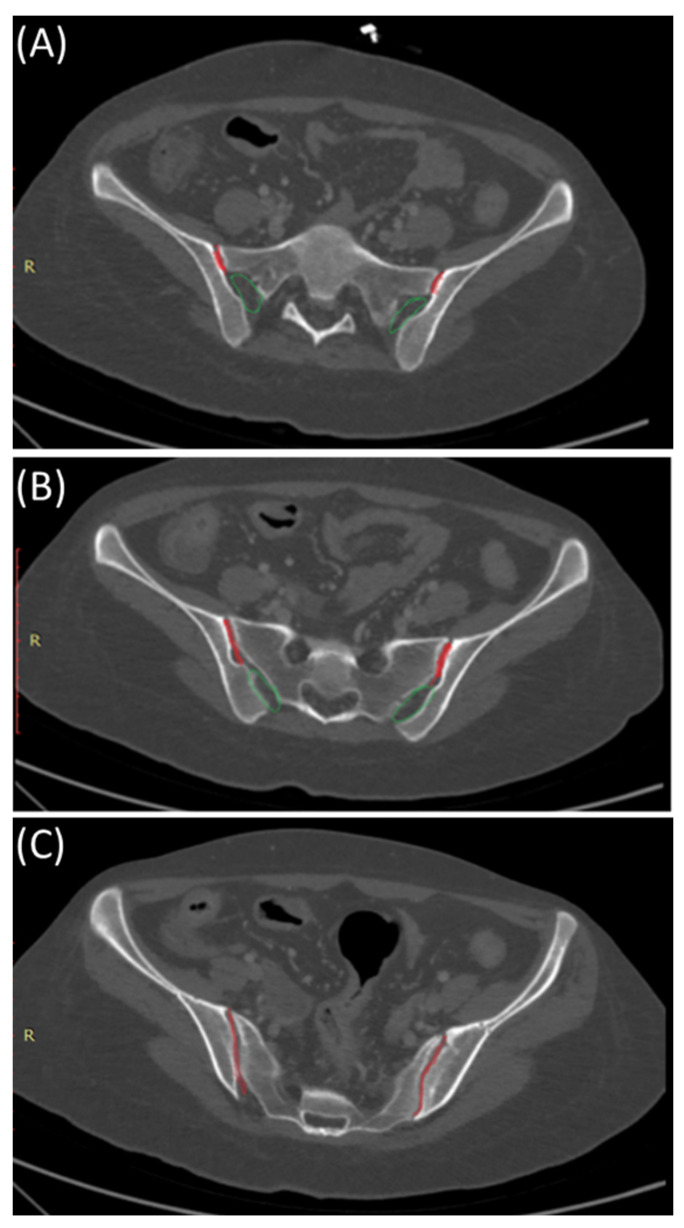
Sacroiliac joint anatomy: Synovial and syndesmotic parts. Axial bone window CT images indicating the position of the synovial (red) and ligamentous (green) components of the sacroiliac joint from cranial (**A**), mid- (**B**), and caudal (**C**) parts of the sacroiliac joints.

The SIJ is the largest axial joint in the body. It is often described as a C-shaped or an auricular-shaped structure [10,11]. As shown in Figure 2, the sacroiliac joint is divided into three parts craniocaudally. In the superior part, the posterior portion is syndesmotic up until the very superior section of the anterior part, where the interosseous sacroiliac ligament connects the sacrum and ilium and merges with the posterior sacroiliac ligament. In the middle part, the posterior section is syndesmotic, whereas the anterior part is synovial, and finally, the inferior part of the joint is identified as a true synovial joint [12,13,14]. These ligamentous connections alongside the muscles surrounding the SIJ limit the joint movement and provide stability [11,14]. There is a wide range of variability in the adult SIJ, including differences in shape and surface contour [14].

The SIJ is considered a diarthrodial joint consisting of the sacrum, forming the concave component, and the ilium, forming the convex component, both of which form an interlocking mechanism with the hyaline cartilage covering the joint surfaces [15]. Moreover, the irregular surface and the wedge shape of the sacrum contribute significantly to the aforementioned mechanism [15].

The SIJ has axes of movement that pass obliquely across the pelvis [15]. Flexing the hip will glide the ipsilateral ilium backward and downwards while extending the hip will glide the ilium forward and away from the sacrum [15]. In addition, the internal oblique and lumbar multifidi muscles stiffen the spine during the spinal motion to reduce interspinal motion [15].

This joint does not have any muscles directly responsible for generating active movements. Instead, muscles primarily influence the motion of the lumbar spine and hip [16]. However, it is surrounded by many muscles that include the psoas, gluteal, hamstrings, erector spinae, quadratus lumborum, abdominal obliques, piriformis and pelvic floor muscles [16].

Its ligaments are the anterior sacroiliac ligament, the interosseous sacroiliac ligament, the posterior sacroiliac ligament, the sacrospinous ligament, and the sacrotuberous ligament. [17]. Additionally, the iliolumbar ligaments have an important role in maintaining SIJ stability [17].

Anteriorly, the SIJ receives innervation from branches originating from the ventral rami of both L4 and L5, either separately or, in some cases, from both [18]. Posterior innervation is provided by a network of lateral branches of the L5-S4 posterior rami, collectively known as the posterior sacral network (PSN) [19].

### 3.2. Clinical Presentation and Etiology of Sacroiliitis

The most common clinical manifestation of sacroiliitis is low back pain, which, generally, affects approximately 70% of individuals at some point in their lives [20]. Moreover, SIJ dysfunction is a major cause of chronic low back pain and may account for up to 20% of low back pain complaints in the general population [21].

This pain can be unilateral or bilateral, with unilateral pain occurring four times more frequently than bilateral pain [21]. The pain in the sacroiliac joint and surrounding structures can manifest itself in various patterns, such as lower back, pelvic, buttock, or sacral pain, as well as discomfort in the groin, posterolateral thigh, and abdomen Additionally, patients may experience acute abdominal pain localized in the lower quadrants and tenderness during rectal examination [22,23]. Athletes often report experiencing lower back pain that makes it challenging to find a comfortable position [24]. In severe cases, patients may remain immobile in bed with their legs extended [21].

Despite the fact that sacroiliitis can manifest in various ways, its presentation is typically classified into acute and chronic [21]. Acute sacroiliitis often mimics other conditions and is frequently overlooked during its initial presentation. New-onset severe pain is a significant clinical symptom of acute sacroiliitis. This pain is typically intense and can be disabling for some patients. It intensifies during activities such as running, climbing stairs, or standing up from a seated position, which can hinder their ability to walk, sit, or even shift in bed [21,24,25,26]. Patients may also describe the pain as numbness, clicking, or popping, typically occurring below the belt line. Additionally, it may radiate to the groin [27].

In the context of acute sacroiliitis, it is widely recognized that pyogenic sacroiliitis is the most frequently reported cause in the medical literature [21]. It typically presents unilaterally and often includes symptoms like a high or low-grade fever and intense, persistent pain originating from the affected sacroiliac joint [22,23]. Proposed factors that may increase the risk of pyogenic sacroiliitis encompass intravenous drug use, infections, especially those originating from skin tissues and infective endocarditis, as well as pregnancy [22,28]. Additionally, there have been documented cases of Clostridium sacroiliitis with gas gangrene occurring after SIJ injections [28]. Nevertheless, it is worth noting that the primary source of infection cannot be pinpointed in more than 40% of patients diagnosed with pyogenic sacroiliitis [22,29].

Another worth-noting entity is acute brucellar sacroiliitis, which is clinically similar to pyogenic sacroiliitis. Bilateral involvement of the sacroiliac joints may be observed in up to 25% of patients. Additionally, healthcare providers should not overlook concomitant clinical signs indicative of brucellosis, such as fever, sweating, malaise, and hepatosplenomegaly [21]. It can either cause septic or reactive sacroiliitis and acute sacroiliitis induced by crystalline deposits in gout and calcium pyrophosphate dehydrate (CPPD) deposition disease, both mimicking the clinical picture of pyogenic sacroiliitis.

SIJ aspiration, guided by ultrasound or CT, is a diagnostic procedure that plays a crucial role in evaluating acute sacroiliitis, particularly when septic or crystal-induced causes are under consideration [13].

The procedure is indicated when there is suspicion of septic sacroiliitis, as it allows for the collection of synovial fluid from the joint for microbiological analysis [30]. This analysis is instrumental in identifying the causative infectious agent, guiding the selection of appropriate antibiotic therapy [30]. Similarly, in cases of crystal-induced sacroiliitis, SIJ aspiration aids in identifying the presence of crystals within the joint fluid, offering diagnostic clarity and guiding the development of targeted treatment strategies [31].

Both ultrasound and CT can be employed to guide the needle placement during SIJ aspiration [13]. Ultrasound provides real-time imaging, particularly advantageous for visualizing soft tissues [32], while CT offers detailed cross-sectional images, valuable in certain clinical contexts [33]. The patient is positioned lying down, and local anesthesia is administered to minimize discomfort. Under the guidance of ultrasound or CT, a thin needle is carefully advanced into the sacroiliac joint space and synovial fluid is aspirated [34].

Dual-energy-computed tomography (DECT) emerges as a pivotal diagnostic tool in the comprehensive evaluation of sacroiliitis [35]. Its dual-energy approach enhances sensitivity, enabling the detection of subtle inflammatory changes such as bone marrow edema and synovial enhancement [36]. Crucially, DECT excels in distinguishing between inflammatory and non-inflammatory alterations within the sacroiliac joints, aiding in the identification of sacroiliitis associated with spondyloarthritis [37]. It is also utilized in detecting nearby tophaceous deposits in conditions such as gout, thus differentiating between septic and crystal-induced sacroiliitis [38].

The aspirated synovial fluid is then subjected to laboratory analysis [39]. In cases of septic sacroiliitis, microbiological cultures are performed to identify the infectious organism [39]. For crystal-induced sacroiliitis, the fluid is examined for the presence of crystals, such as monosodium urate or calcium pyrophosphate crystals [40]. Positive findings in the synovial fluid analysis confirm the diagnosis of septic or crystal-induced sacroiliitis, providing critical information for targeted and effective treatment [41].

Moreover, rarely, acute sacroiliitis can be associated with isotretinoin treatment, a retinoid prescribed for severe acne, which has been linked to various musculoskeletal side effects [21]. These side effects may include arthralgia with or without arthritis, myalgia, and soft tissue calcification. Sacroiliitis typically emerges within days or weeks after the initiation of isotretinoin treatment, presenting with symptoms such as sacroiliac pain, peripheral arthritis, and, on occasion, the development of muscle weakness or poorly understood neuropathy [21].

In the context of chronic sacroiliitis, the disease is considered a hallmark of ankylosing spondylitis [21] and it is also observed in other spondyloarthropathies such as psoriatic arthritis, reactive arthritis, and enteropathic arthritis as an extraintestinal manifestation of ulcerative colitis or Crohn’s disease [1]. Additionally, the prevalence of chronic sacroiliitis seems to be higher in patients with familial mediterranean fever (FMF) compared to the general population [1]. Furthermore, although its incidence is low, tuberculosis can penetrate into the cancellous bone and joint synovium and cause tuberculous sacroiliitis [42].

### 3.3. Radiological Findings and Imaging Modalities

Radiological imaging plays a crucial role in diagnosing and evaluating sacroiliitis [43]. The radiological features of sacroiliitis can be observed using several imaging modalities, including X-rays, CT scans, and MRIs and Bone Scans [13].

#### 3.3.1. Plain Radiographs

**Figure 3:**
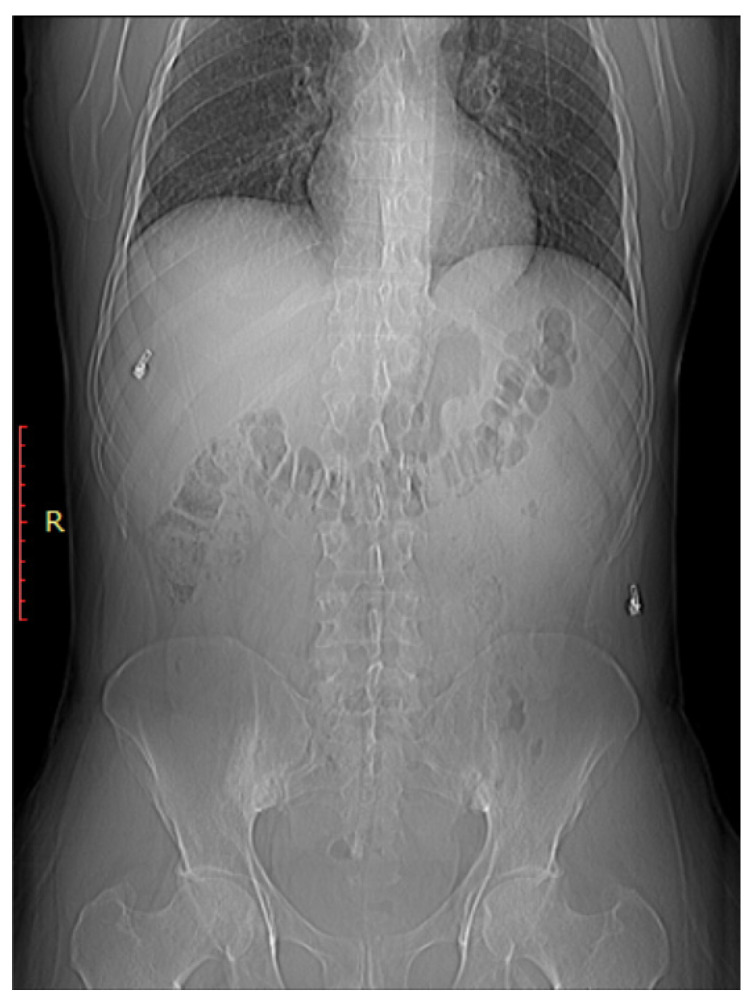
Bilateral sacroiliitis. X-ray for a 41-year-old male presenting with Rt sciatica showing irregularity and sclerosis of both sacroiliac joints is more evident on the right.

As shown in Figure 2, early sacroiliitis may show subtle erosions at the joint margins [44]. These erosions are typically irregular and may appear as small, well-defined areas of bone loss [44]. As the inflammation progresses, the joint may become more sclerotic, leading to increased bone density in the SI joint [45]. Progressive inflammation can lead to narrowing of the SIJ space, indicating joint damage and dysfunction As shown in Figure 3, early sacroiliitis may show subtle erosions at the joint margins [44]. These erosions are typically irregular and may appear as small, well-defined areas of bone loss [44]. As the inflammation progresses, the joint may become more sclerotic, leading to increased bone density in the SI joint [45]. Progressive inflammation can lead to narrowing of the SIJ space, indicating joint damage and dysfunction [1]. In addition, changes in the subchondral bone, including irregularities, sclerosis, and erosions, are common features of sacroiliitis [46].

In severe cases or in advanced stages of ankylosing spondylitis (a type of inflammatory arthritis), the sacroiliac joints may become completely fused or ankylosed. On X-ray, this presents as a loss of joint space and a bony bridge or fusion between the sacrum and ilium [1].

X-rays may not be sensitive enough to detect early or subtle changes in sacroiliitis, especially in cases of non-radiographic axial spondyloarthritis [47]. In such cases, other imaging modalities like MRI are often preferred for their higher sensitivity in detecting active inflammation and early disease [47].

#### 3.3.2. Computed Tomography

**Figure 4:**
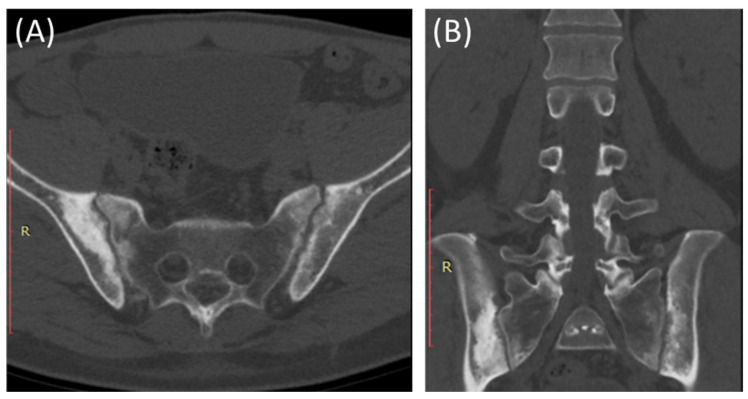
Axial bone window CT scan (**A**) and coronal bone window CT scan (**B**) for a 41-year-old male presenting with Rt sciatica suggestive of bilateral sacroiliitis (same Patient as in Figure 3).

**Figure 5:**
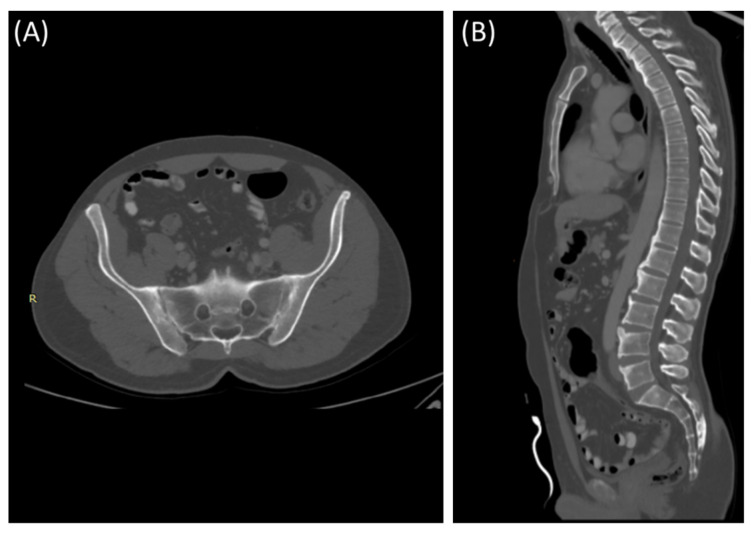
Axial bone window CT scan (**A**) showing Chronic sacroiliitis with ankyloses of the sacroiliac and a sagittal bone window CT scan (**B**) showing Syndesmophytes in the spine in a 55-year-old patient with Ankylosing spondylitis.

CT scans provide more detailed images of the sacroiliac joint and can reveal erosions, sclerosis, and joint space narrowing with greater clarity than X-rays [13]. Severe sacroiliitis, particularly in cases of ankylosing spondylitis, can lead to complete fusion or ankylosis of the sacroiliac joint [4]. CT scans can demonstrate the fusion as a solid, bony bridge between the sacrum and ilium [4]. CT scans can also assess soft tissues surrounding the SIJ, such as ligaments, tendons, and muscles, which may be inflamed in cases of sacroiliitis [44] as demonstrated in Figure 4 and Figure 5.

CT scans can provide detailed information about adjacent structures, such as the lumbar spine, hip joints, and pelvic bones, to evaluate for any associated abnormalities or complications [48].

#### 3.3.3. Magnetic Resonance Imaging

MRI is considered the gold standard for the diagnosis of sacroiliitis, especially in early and inflammatory stages [5]. It provides valuable information for diagnosing and monitoring the condition, assessing disease activity, and guiding treatment decisions [5].

As demonstrated in Figure 4, MRI is particularly sensitive in detecting active inflammation [44]. In sacroiliitis, MRI often reveals edema (swelling) and increased signal intensity on T2-weighted images within and around the SIJ [13]. This edema is indicative of active inflammation and is a hallmark feature [13].

Bone marrow edema refers to increased water content within the bone marrow, typically seen as high signal intensity on T2-weighted images [49]. It is a key finding in early sacroiliitis and indicates active disease [49].

MRI can visualize changes in the SIJ space, including joint space widening or narrowing [50]. Widening may be seen in the acute phase due to joint effusion, while narrowing indicates chronic damage [50].

MRI can detect erosions or bone defects at the edges of the SIJ [49]. These erosions are usually more visible on T1-weighted images and may appear as areas of low signal intensity [51]. Areas of sclerosis, where the bone becomes denser due to chronic inflammation, may be visible on MRI as regions of low signal intensity on T2-weighted images [52].

**Figure 6:**
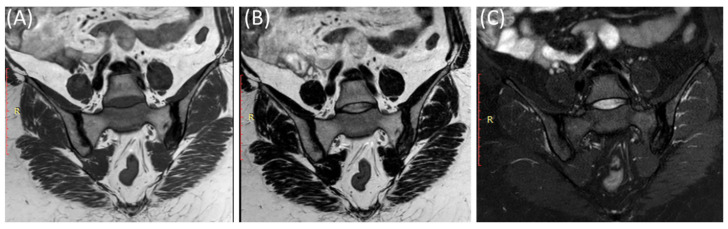
Coronal T1-WI image (**A**), coronal T2-WI image (**B**) and coronal T2-fat suppression image (**C**) showing sclerosis and mild irregularity in both sacroiliac joints, more on the left side with no evident bone marrow edema in a 52-year-old female with chronic low back and hip pain suggestive of bilateral chronic sacroiliitis.

MRI can reveal soft tissue changes, including inflammation of the ligaments and tendons around the SIJ [50]. Enthesitis, which is the inflammation of the insertion points of ligaments and tendons into bone, is a common finding in sacroiliitis and can be visualized [53]. Furthermore, a contrast agent (gadolinium) may be used to enhance areas of active inflammation, making them more visible [54]. In advanced cases, particularly in ankylosing spondylitis (Figure 6), MRI may show evidence of joint ankylosis, where the sacroiliac joint becomes fused [44].

**Figure 7:**
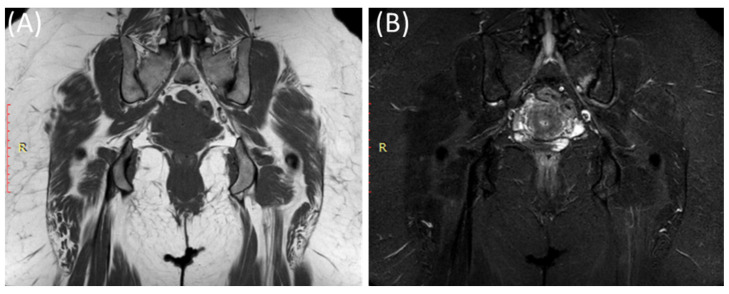
Coronal T1WI image (**A**) and STIR image (**B**) showing edema and irregularity in the left sacroiliac joint in a 47-year-old female with lower back pain and left sciatica with acute (active) sacroiliitis.

As shown in Figure 7, fat suppression techniques or fat saturation sequences are often used in MRI to suppress the signal from fat tissue, making it easier to identify areas of inflammation and edema [55,56].

In axial spondyloarthritis (axSpA), early synovitis in the sacroiliac joints can be accessed via MRI, revealing key features indicative of inflammatory changes [57]. One notable finding is bone marrow edema, which manifests as increased signal intensity on T2-weighted and STIR sequences [58]. Additionally, synovial hypertrophy may lead to joint space widening and increased fluid within the joint, highlighting the presence of inflammation [59]. Soft tissue inflammation, including synovitis, may also be apparent in the vicinity of the SI joints [59]. While erosive changes in the subchondral bone may develop over time, these are more characteristic of later disease stages [60]. The pattern of involvement often exhibits asymmetry in the sacroiliac joints, distinguishing it from conditions like rheumatoid arthritis [61].

#### 3.3.4. Nuclear Medicine—Bone Scans

Nuclear medicine scans (bone scans) can also play a role in the evaluation of sacroiliitis, particularly in detecting active inflammation and assessing disease activity and inflammatory changes earlier than conventional X-rays [6,7].

Those scans can show the extent of inflammation and involvement of multiple joints in cases of spondyloarthritis [62]. Bone scans can provide a whole-body assessment, which is valuable for identifying other areas of inflammation or disease activity in conditions like ankylosing spondylitis [62].

**Figure 8:**
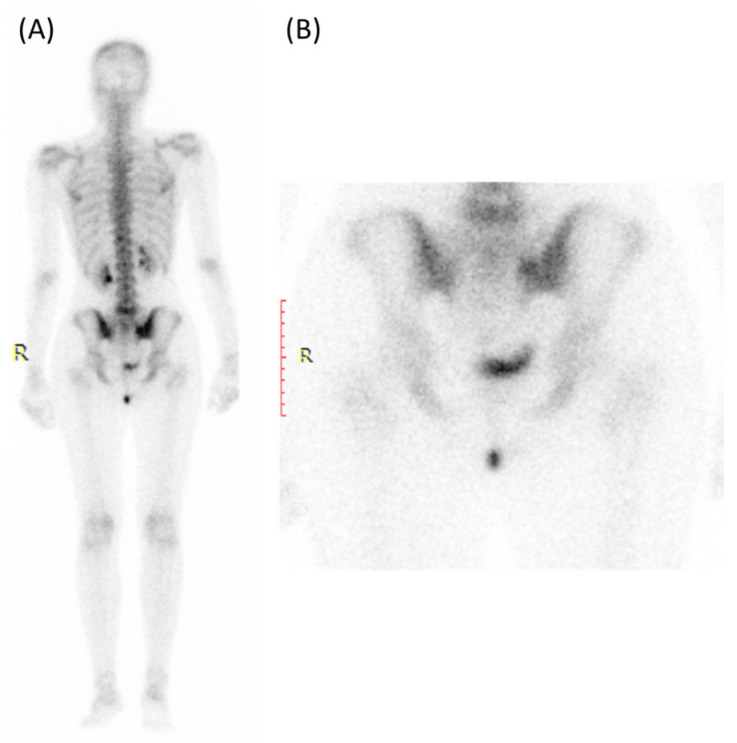
Bone scan (using Tc99m HDP) for a 30-year-old female presented with Lt hip pain showing focal increased tracer uptake in the Lt sacroiliac joint in the blood pool (**A**) and delayed images (**B**).

As provided in Figure 8, one of the most commonly used nuclear medicine scans for sacroiliitis is the Technetium-99m bone scan [6]. This tracer is taken up by bone tissue in areas of increased metabolic activity [6]. In sacroiliitis, areas of active inflammation and increased bone turnover will absorb more of the radioactive tracer, leading to “hot spots” on the scan [63]. A bone scan can provide information about the extent and distribution of inflammation in the sacroiliac joints and other areas of the body, if applicable [64]. It is particularly useful for detecting active inflammation and assessing the overall disease activity in sacroiliitis [6].

However, bone scans are not specific and they do not provide detailed anatomical images, so they are often used in conjunction with other imaging modalities like X-rays or MRI [65]. Also, the radioactive tracer used has a relatively short half-life, so the timing of the scan is critical to capture the active disease process [66]. Moreover, radiation exposure is a consideration, even though the dose is typically low and safe [67].

### 3.4. Radiological Criteria and Scoring Systems

Sacroiliitis is graded using plain films based on the Modified New York Criteria in which the sacroiliac joint is observed and graded as one of five different grades. Grade 0 is given to a normal sacroiliac joint; Grade 1 is given if there is observed suspicion (blurriness surrounding the margins of the joint); Grade 2 if there are small abnormalities, including restricted sclerosis or erosions; Grade 3 if there are explicit and extensive anomalies including changes in the width of the joint, and a significant number of erosions; finally, Grade 4 would be given if complete ankylosis is observed. It is worth mentioning that the diagnosis of sacroiliitis depends on its laterality. According to modified New York criteria, if there is bilateral sacroiliac involvement, the lesions observed should be at least Grade 2 for a diagnosis to be achieved. If the involvement is unilateral, the lesions observed should be at least Grade 3.

The Spondyloarthritis Research Consortium Canada (SPARCC) MR-based scoring system relied on T2-weighted images, as inflammation-induced edema around the marrow might not be visible in other phases, and fat signal suppression in the marrow can be seen in T2 as well [68]. Each sacroiliac joint was divided into four quadrants, giving one point to every quadrant demonstrating an increased STIR signal [68]. An additional point would be given for any joint demonstrating an intense signal, and another for a continuous signal that is more than one centimeter in depth [68]. This is then applied to six coronal slices [68]. Each slice would have 12 points maximum, and the total amount of points for all six slices would be 72 points [68].

Other MR-based scoring systems include MR Imaging of Seronegative SpA (MISS), Leeds, Aarhus, a system proposed by Sieper and Rudwaleit, and another by Hermann and Bollow when applying the Outcome Measures in Rheumatology (OMERACT) filter on mentioned scoring systems, where 52% voted that sacroiliac joint MRI could serve as a valuable measure to be used in pharmaceutical clinical trials, while only 30% agreed that they are valuable in evaluating chronic changes for spine and sacroiliac assessment

Despite radiological criteria, fluoroscopically guided sacroiliac-joint block injections remain the most reliable method for diagnosing pain originating from within the sacroiliac joint. A single block or a dual block approach could be used, with the dual block approach using lidocaine first, followed by bupivacaine. The test is deemed positive if at least a 70% relief of pain is achieved for dual block, and 75% for single block. Pain relief of at least 50% indicates that the pain is highly influenced by a sacroiliac joint condition [20,69].

### 3.5. Management of Sacroiliitis

A patient with sacroiliitis should be approached by first identifying and addressing potential contributing factors, including hip osteoarthritis, kyphosis, scoliosis, leg length discrepancy, and osteoporosis. Subsequently, education and management can be initiated via a home exercise program, activity modification, and physical therapy. Lastly, pain management can be considered to maintain and facilitate activity, either via pharmacological interventions or complementary and alternative modalities (CAM) [70]. After 6 weeks, if a reduction in pain of approximately 30 percent and an improvement in function are observed, the used treatment and self-assessment plan should be continued. If not, the option of escalating pharmacological therapy or discussing the possibility of sacroiliac joint or periarticular corticosteroid injection with the patient, provided there are no contraindications, should be considered. At this point, local anesthesia and corticosteroid injections should be administered if there is less pain and the benefits are sustained for at least 3 months. The injection should be repeated every 12 weeks, with a maximum limit of three injections within a 12-month period, while also continuing self-management practices [22,71,72].

If an improvement with local corticosteroids and local anesthesia is not sustained for 3 months, consideration can be given to discussing an alternative treatment plan, such as denervation, unless it is declined by the patient. In the event of the patient declining this option, a reevaluation of CAM and a stepped-care drug assessment for sacroiliac joint pain should be contemplated. If pain relief is not attained with local analgesia and corticosteroid injections, a reevaluation of the stepped-care drug assessment and CAM should be undertaken. If improvement still does not occur, a reconsideration of injection pathways or a referral to an interdisciplinary pain rehabilitation program may be warranted [70].

Ultimately, the approach to sacroiliitis management should be individualized, considering the patient’s unique circumstances and responses to various treatments. Regular reassessment and adjustments to the treatment plan, including considerations of alternative therapies and interdisciplinary pain rehabilitation programs, may be necessary for those who do not achieve satisfactory relief with initial interventions. The goal remains to improve the patient’s quality of life and alleviate the discomfort associated with sacroiliitis while minimizing the need for invasive procedures whenever possible [22,71,72].

### 3.6. Emerging Technologies and Future Perspectives

Many studies revealed techniques that offer high-resolution imaging, which can aid in the early detection and assessment of disease activity [42]. Among these techniques is dual energy-computed tomography (CT), which differs from conventional CT in that it involves the attainment of images at two different energy levels; therefore, it can differentiate between parts with high and low atomic numbers [73,74]. Furthermore, it is considered a great alternative for patients for whom MRI is contraindicated [73,74]. Moreover, three-dimensional MRI sequences (high-resolution sequences), ameliorate the visualization of structural changes in the sacroiliac joint such as erosions owing to the high contrast between joint components [75]. Furthermore, 3D printing technology is used to investigate the creation of anatomical models for surgical planning and educational purposes. This technology can help surgeons plan interventions and better understand the complex anatomy of the sacroiliac joints [76,77].

In addition, potential therapies for sacroiliitis regenerative medicine, including platelet-rich plasma (PRP) injections, and stem cell therapy are being explored as potential treatments for sacroiliitis. They are supposed to stimulate tissue repair and lessen inflammation [78].

Evidence revealed the effectiveness of Tumor Necrosis Factor (TNF) inhibition in the treatment of sacroiliitis especially that associated with inflammatory conditions such as ankylosing spondylitis and other spondyloarthritis. They proved to be a milestone in the treatment of this condition as they affect disease activity, function, and life quality of patients with these conditions [79]. Additionally, Janus Kinase inhibitors (JAK), which are targeted synthetic disease-modifying anti-rheumatic drugs (DMARDs), are now recommended for patients who failed ≥2 NSAIDs and also have either the elevated C reactive protein, MRI inflammation of sacroiliac joints or radiographic sacroiliitis, and have an Ankylosing Spondylitis Disease Activity Score of more than or equal to 2.1 [80,81].

The integration of artificial intelligence (AI) in the realm of sacroiliitis holds considerable promise across various facets of disease management [82]. One key application lies in diagnostic imaging, where AI algorithms can meticulously analyze X-rays, CT scans, and MRIs to identify and quantify indicators of sacroiliitis, including subtle changes in the sacroiliac joints and inflammatory markers like bone marrow edema [82]. Furthermore, AI can enhance efficiency by automating the generation of diagnostic reports, alleviating the workload on radiologists and potentially improving the report consistency [83].

In the realm of early detection and prediction, AI models prove valuable in risk stratification, identifying individuals at a heightened risk of developing sacroiliitis [84]. These models can also predict disease progression based on clinical and imaging data, allowing for timely intervention and personalized treatment approaches [84]. AI’s role extends to treatment optimization, where algorithms can predict individual patient responses to specific therapies, guiding clinicians in selecting the most effective interventions [85].

In the research domain, AI’s capacity for large-scale data analysis proves instrumental [86]. By scrutinizing extensive datasets, including electronic health records and imaging archives, AI identifies patterns, trends, and potential biomarkers associated with sacroiliitis, contributing to a more profound understanding of the disease and the development of novel therapeutic avenues [82].

A study by Lee et al. utilized AI to develop a diagnostic tool for more accurate detection of sacroiliitis in radiological images [87]. The AI model demonstrated high accuracy for different sacroiliitis grades, with percentages ranging from 94.53% to 98.44%, and achieved 100% sensitivity for Grade 3 and normal cases, as well as 100% specificity for Grade 4.

## 4. Conclusions

In this review, we delved into sacroiliitis, an inflammatory condition affecting the sacroiliac joint. This condition can manifest acutely or chronically, causing lower back pain and discomfort. Diagnosis relies heavily on radiological techniques, with MRI being the gold standard due to its sensitivity in detecting early and inflammatory changes.

The management of sacroiliitis varies, ranging from conservative approaches like physical therapy and medication to interventional treatments such as joint injections and radiofrequency denervation. Surgical interventions, including joint fusion, can be considered in severe cases.

The future of sacroiliitis management holds promise with emerging technologies like high-resolution imaging and regenerative medicine. Biologic therapies have also transformed the treatment landscape for individuals with inflammatory conditions like ankylosing spondylitis, significantly improving their quality of life.

## Data Availability

All data are available in the presented manuscript.
